# Infection control management and surveillance of carbapenem-resistant Gram-negative bacteria in hematopoietic stem cell recipients

**DOI:** 10.1186/s13756-019-0606-3

**Published:** 2019-10-21

**Authors:** Claas Baier, Maleen Beck, Viktoria Panagiota, Catherina Lueck, Daniel Kharazipour, Sophie Charlotte Hintze, Robin Bollin, Ella Ebadi, Stefan Ziesing, Matthias Eder, Franz-Christoph Bange, Gernot Beutel

**Affiliations:** 1 Institute for Medical Microbiology and Hospital Epidemiology, Hannover Medical School, Carl-Neuberg-Straße 1, 30625 Hannover, Germany; 2 Department of Hematology, Hemostasis, Oncology and Stem Cell Transplantation, Hannover Medical School, Hannover, Germany; 3 Department of Gastroenterology, Hepatology and Endocrinology, Hannover Medical School, Hannover, Germany; 4 Clinic for Nephrology, Hannover Medical School, Hannover, Germany

**Keywords:** Carbapenem resistance, Gram-negative bacteria, Colonization, Bloodstream infection, Screening, Hematopoietic stem cell transplantation, Infection control

## Abstract

Hematopoietic stem cell transplantation (HSCT) is a curative treatment option for selected diseases of the hematopoietic system. In the context of HSCT, bloodstream infections caused by Gram-negative bacteria (GNB) significantly contribute to morbidity and mortality. Antibiotic treatment of bloodstream infections with carbapenem-resistant (CR) GNB presents a particular challenge. As a part of our infection control management, the admission of a patient who was known to be colonized with a CR *Acinetobacter baumannii* triggered an active weekly screening of all patients to determine the prevalence and potential transmission of CR GNB and CR *Acinetobacter baumannii* in particular. Over a 3 month period a total of 71 patients were regularly screened for colonization with CR GNB. Including the index patient, a total of three patients showed CR GNB colonization representing a prevalence of 4.2%. Nosocomial transmission of CR *Acinetobacter baumannii* or other CR GNB was not observed. However, the index patient developed a subsequent bloodstream infection with the CR *Acinetobacter baumannii*, therefore empiric antibiotic therapy based on the known resistance profile was initiated. A weekly prevalence screening for CR GNB might be an effective monitoring tool for potential transmission, may enhance existing infection control management concepts and may support the decision making for empiric antibiotic therapy.

## Background

Patients undergoing hematopoietic stem cell transplantation (HSCT) are at high risk for nosocomial infections. One possible mechanism is that conditioning regimens induce leaky gut syndrome and promote translocation of Gram-negative bacteria (GNB) from the gut and thus cause bloodstream infections (BSIs) [1]. Furthermore, the antibiotic therapy in those patients has recently become more difficult since the emergence of carbapenem-resistant (CR) GNB [2]. CR GNB are typically resistant to first-line antibiotic therapy agents which are recommended by guidelines for empiric antibiotic therapy for fever in neutropenia [3]. In hematopoietic stem cell recipients and neutropenic patients an increased mortality has been shown in case of a BSI caused by CR GNB [4, 5].

As a part of our infection control management, we closely monitored the prevalence of CR GNB on our HSCT unit, while an index patient with a CR *Acinetobacter baumannii* was treated on the ward. Within this report we discuss the implications of CR GNB for infection control and guidance of empiric antibiotic therapy.

## Methods

The Hannover Medical School is a 1500-bed university hospital in northern Germany. The HSCT unit consists of 6 two-bed and 8 single-bed rooms with en-suite bathrooms, anterooms and high-efficiency particulate air filtration with positive pressure. The unit specifically admits patients undergoing autologous and allogeneic stem cell transplantation, as well as those suffering from associated complications (e.g. graft-versus-host-disease or severe infections). During conditioning and until hematologic recovery patients are individually housed. Professional clothing and surgical masks are mandatory for visitors and healthcare workers (HCWs) whenever entering the patient’s room. Pre-transplant colonization surveillance of the patients admitted to the HSCT unit included screening for Methicillin-resistant *Staphylococcus aureus* (MRSA), GNB (including CR ones), Vancomycin-resistant enterococci (VRE) and *Candida species.* For the MRSA screening a nasopharyngeal swab was taken and plated on Brilliance MRSA 2 Agar (Oxoid, Wesel, Germany). For GNB, VRE, and *Candida species* rectal swabs or stool samples were plated on an in-house produced MacConkey agar without antibiotics and supplemented either with cefotaxime or ceftazidime at final concentrations of 1 mg/L, on CHROMagar™ mSuperCARBA™ (CHROMagar, Paris, France) to detect carbapenem resistance, on Brilliance VRE Agar (Oxoid, Wesel, Germany), and on an in-house produced malt extract agar. As in winter/spring 2019 a patient with known colonization with a CR *Acinetobacter baumannii* was admitted, the surveillance was extended by a weekly prevalence screening for CR GNB over a 3 month period. For this specific screening rectal and groin swabs were taken weekly from every inpatient on the ward. The CR GNB screening swabs were plated on a selective culture media (CHROMagar™ mSuperCARBA™, CHROMagar, Paris, France) and cultured according to the manufacturer’s instructions. Species identification was usually done by the Matrix Assisted Laser Desorption Ionization Time-of-Flight method (VITEK® MS, bioMérieux, Marcy-l’Étoile, France). The VITEK® 2 system (bioMérieux, Marcy-l’Étoile, France) was used for initial antimicrobial susceptibility testing. Testing included meropenem and ertapenem for *Enterobacterales,* and meropenem and imipenem for *Acinetobacter baumanii*. *Enterobacterales* which i) had an increased minimal inhibitory concentration (MIC) for meropenem, and/or ii) were categorized as resistant or intermediate for ertapenem and did not harbor an intrinsic AmpC (e.g. *Escherchia coli*), were subsequently tested in the Merlin system (Merlin Diagnostika, Bornheim-Hesel, Germany) for meropenem, imipenem and ertapenem. They were also tested for the presence of carbapenemases. *Acinetobacter baumannii* isolates categorized as resistant or intermediate for meropenem and/or imipenem in the VITEK® 2 system were subsequently tested in the Merlin system for meropenem and imipenem. Susceptibility testing categories were not edited if a carbapenemase was detected, but the presence of the carbapenemase was reported. For categorization of susceptibility (“S” = susceptible, “I” = intermediate, “R” = resistant) we followed the recommendation of the European Committee on Antimicrobial Susceptibility Testing (EUCAST). GNB categorized as “I” or “R” for meropenem and/or imipenem and GNB which harbored a carbapenemase were here defined as CR. The new nomenclature of the “I” category as “susceptible, increased exposure”, introduced by EUCAST in 2019, was not yet used in the laboratory during the study period. In *Enterobacterales* carbapenemases were tested using immunochromatography (RESIST-4 O.K.N.V. for carbapenemase producing *Enterobacterales,* targeting OXA-48, KPC, NDM and VIM; Coris BioConcept, Gembloux, Belgium) and nucleic acid amplification (Xpert® Carba-R for carbapenemase producing organisms, targeting OXA-48, KPC, NDM, VIM and IMP-1; Cepheid, Sunnyvale, California, USA). For *Acinetobacter baumannii* immunochromatography (OXA-23 K-SeT targeting OXA-23; Coris BioConcept, Gembloux, Belgium) and the Xpert® Carba-R system (see above) were used. For the molecular investigation of the bacterial isolates a pulsed-field gel electrophoresis (PFGE) was performed according to an in-house protocol (1% agarose gel, restriction enzyme Spe I for *Escherichia coli*).

## Results

During the observation period a total of 71 patients (30 female) representing 75 inpatient stays were on the ward. The mean age was 50 years and the mean length of stay was 19 days. The most prevalent underlying neoplasia was acute myeloid leukemia which occurred in a total of 28 (39.4%) patients. Twenty-two patients received an allogeneic and 2 an autologous HSCT during the observation period. Overall, 316 specimens from the prevalence screening were collected (157 rectal swabs and 159 groin swabs). Three patients with CR GNB were detected which accounts for a cumulative prevalence of 4.2%. The species found were *Acinetobacter baumannii* (*n* = 1) and *Escherichia coli* (*n* = 2). Two patients had been known already as carriers of CR GNB, one of whom was colonized with *Acinetobacter baumannii* and the other with *Escherichia coli.* In both patients the respective CR GNB was found on admission and in several specimens of the weekly CR GNB screening. In a third patient a CR *Escherichia coli* was found during the weekly screening for the first time. The patient with the CR *Acinetobacter baumannii* colonization developed subsequently a BSI with this *Acinetobacter*, whereas the other two patients did not develop an invasive infection with their CR GNB. Further details on the patients and the CR GNB including phenotypic susceptibility pattern are presented in Table 1. The two CR *Escherichia coli* isolates showed a different PFGE pattern according to the criteria suggested by Tenover et al. [6].

**Table 1:** Characteristics of the patients with CR GNB

Case	Species	Antibiotic resistance phenotype^a^	Carbapenemase^b^	First acquisition on the HSCT unit	Colonization	Subsequent infection with the colonizing CR GNB	Antibiotic treatment of infection	Decolonization procedure
1	*Escherichia coli*	Piperacillin/Tazobactam: R Ceftazidime: R Fluroquinolones: R Gentamicin: R Meropenem: I (8) Ertapenem: R (> 2) Imipenem: I (4) Colistin: S Ceftazidime/Avibactam: S Ceftozolan/Tazobactam: S	Not detected	Yes (after HSCT)	Yes (rectal)	No	–	No
2	*Escherichia coli*	Piperacillin/Tazobactam: R Ceftazidime: R Gentamicin: S Fluroquinolones: R Meropenem: S (0,5) Ertapenem: R (2) Imipenem: S (≤1) Colistin: S Ceftazidime/Avibactam: S Ceftolozan/Tazobactam: S	OXA-48	No, already known at admission (prior to HSCT)	Yes (rectal, groin)	No	–	No
3	*Acinetobacter baumannii*	Piperacillin/Tazobactam: R Ceftazidime: R Gentamicin: R Fluroquinolones: R Meropenem: R (> 16) Imipenem: R (> 8) Colistin: S Ceftazidime/Avibactam: R Ceftozolan/Tazobactam: R	Not detected	No, already known at admission (prior to HSCT)	Yes (rectal, groin, naso-pharyngeal^c^)	Yes (BSI), onset prior to HSCT	High dose meropenem, amikacin, colistin	Yes: Decolonization of skin, oral and nasal mucosa with octenidine-dihydrochloride

^a^Minimal inhibitory concentrations (Merlin system) for the carbapenems are shown in the brackets, the unit is mg/L. ^b^ In the *Escherichia coli* isolates OXA-48, KPC, NDM, VIM and IMP-1 were tested. In the *Acinetobacter baumannii* isolate OXA-23, OXA-48, KPC, NDM, VIM and IMP-1 were tested. ^c^The nasopharyngeal specimen was not part of the regular CR GNB screening. *CR* carbapenem-resistant, *GNB* Gram-negative bacteria, *HSCT* Hematopoietic stem cell transplantation, *BSI* Bloodstream infection, *R* resistant, *I* intermediate, *S* susceptible

In case of CR GNB positivity additional infection control interventions included gloves and gowns for visitors, HCWs and support staff. In parallel, patients with CR GNB and their visitors were trained beyond the usual measures to comply to hand hygiene.

The pre-transplant colonization surveillance on or shortly prior to admission showed one patient with MRSA (1.4%), four with VRE (5.6%) and 5 patients with GNB showing resistance to aminopenicillins and 3rd generation cephalosporins (7.0%).

## Discussion

The CR GNB colonization prevalence in our study cohort was 4.2%. In comparison to some other HSCT units, this rate was relatively low: In a study from Turkey the prevalence was 11.4% [7]. Another study from Brazil even showed that 27% of the patients were colonized by at least one CR GNB [1]. Our CR GNB colonization prevalence was more consistent with that reported in patients with hematologic neoplasms from several centers in Italy (3.8%) [8]. However, beyond hematology and oncology, the overall prevalence of CR GNB in Germany is considerably lower, for instance in a university medical center it was 0.22% in all inpatients [9]. This is supported by data of the German national surveillance system for antimicrobial resistance at the Robert Koch Institute (ARS, data accessible under https://ars.rki.de), that lists a meropenem resistance among *Escherichia coli* isolates of < 1% in 2018. Thus, compared to the national data, the prevalence on our HSCT unit was higher. However, the patient population on a HSCT unit has typical risk factors for CR GNB carriage such as chemotherapy use, multiple hospital stays [7] or previous antibiotic therapy, which at least partly might explain this finding. It is likely that the CR GNB prevalence in HSCT units correlates roughly with the country specific prevalence as published in a multicenter study by the Infectious Diseases Working Party of the European Bone Marrow Transplantation Group [4]. Furthermore, studies in HSCT recipients showed that a previous colonization with multidrug-resistant GNB (including CR ones) is a risk factor for the detection of the respective pathogens in BSI [1, 10, 11].

In our cohort the patient with the known CR *Acinetobacter baumannii* colonization developed subsequent BSI with this pathogen and we assumed a translocation from the gut or nasopharyngeal mucosa during barrier disturbance. Therefore, the initiated empirical antibiotic treatment considered the resistance profile of the colonizing strain and was initiated immediately. Colistin was the main component of the therapy and the patient survived this BSI episode. We would suggest keeping antimicrobial susceptibility testing results in mind for antibiotic stewardship purposes in general, and for treatment escalation in case of persisting fever in HSCT recipients in particular. The weekly prevalence screening detected one patient with a probably nosocomially acquired CR *Escherichia coli* that would have not been found otherwise. However, we assume that this CR *Escherichia coli* was not acquired due to transmission from the already known other patient with a CR *Escherichia coli*. This is because the two CR *Escherichia coli* isolates had different phenotypic resistance patterns and only the isolate of the already known carrier expressed a carbapenemase. Moreover, the PFGE patterns of these two isolates were different, indicating that the CR *Escherichia coli* isolates had not been transmitted. Noteworthy, the patient with the presumed nosocomial acquisition of the CR *Escherichia coli* received a carbapenem therapy prior to the finding, so we suspect selective pressure to play a role here. Moreover, this patient was already more than 2 weeks on the ward prior to the positive result and screenings had been negative for CR *Escherichia coli* on two occasions while on the ward. Due to the limited sensitivity of rectal screenings, however, it cannot be ruled out completely that the patient might had already been colonized on admission with the CR *Escherichia coli*.

Taken together, we demonstrated by the weekly prevalence screening that none of the CR GNB present was transmitted from one patient to another. This shows the effectiveness of our infection control management for CR GNB, which takes into account national and international guidelines [12, 13]. Therefore, we are convinced that patients with CR GNB can be treated on a HSCT unit when effective infection control measures are implemented and strictly followed. Taken together, the cornerstones of our concept were:

i) screening for multidrug resistant bacteria (including CR GNB) prior to or no later than on admission,
ii) strict contact precautions and single room isolation for CR GNB carriers,
iii) emphasis of hygienic hand disinfection for patients, visitors, HCWs and support staff,
iv) weekly prevalence screening for CR GNB for all patients if an index patient with CR *Acinetobacter baumannii* or CR *Klebsiella pneumoniae* is on the ward,
v) octenidine-dihydrochloride based skin and nasopharyngeal mucosa decolonization for carriers of CR *Acinetobacter baumannii* and CR *Klebsiella pneumoniae*, and
vi) enhanced terminal wipe-disinfection for the patient room after discharge.

Implementation and practical applicability of our screening approach was well accepted by patients and HCWs.

However, we are aware that this analysis has some limitations. Larger prospective studies are necessary to further evaluate the value of a weekly screening of all patients on the ward in the presence of one index patient with CR *Acinetobacter baumannii* or CR *Klebsiella pneumoniae*. Especially the question in how far such a screening can be an effective tool for monitoring transmission is of relevance. Moreover, these studies might address for example the effect of a weekly screening on the incidence of CR GNB or the use of reserve antibiotics such as colistin. This applies even more for a general (weekly) prevalence screening with regard to CR GNB. The patients in this analysis were treated in a single HSCT unit, located in a country with comparatively low prevalence for CR GNB. Therefore, our results do not necessarily apply to other hospitals and settings. Moreover, the number of patients included was limited by the short study period of 3 months. We took samples from two different anatomical sites (groin and rectal) for the weekly prevalence screening. Thus sensitivity of detection of *Acinetobacter baumannii* might have been somewhat limited, and an additional extended cutaneous sampling or pharyngeal samples might have further increased the sensitivity of screening [14, 15].

In conclusion, the knowledge of CR GNB in terms of colonization and infection is crucial to guide infection control measures and empiric anti-infective therapy in HSCT recipients. A weekly CR GNB prevalence screening might be effective in monitoring potential transmissions and may offer the potential to enhance admission screening, but further studies are needed in this regard.

## Data Availability

All data generated or analysed during the current study are included in this published article.

## Abbreviations

BSI: Bloodstream infection
CR: Carbapenem-resistant
GNB: Gram-negative bacteria
HCW: Healthcare worker
HSCT: Hematopoietic stem cell transplantation
MIC: Minimal inhibitory concentration
MRSA: Methicillin-resistant *Staphylococcus aureus*
PFGE: Pulsed-field gel electrophoresis
VRE: Vancomycin-resistant enterococci
